# miR-140-3p is involved in the occurrence and metastasis of gastric cancer by regulating the stability of FAM83B

**DOI:** 10.1186/s12935-021-02245-8

**Published:** 2021-10-16

**Authors:** Zhengguang Wang, Ke Chen, Dongchang Li, Mengding Chen, Angqing Li, Jian Wang

**Affiliations:** 1 Department of General Surgery, The First Affiliated Hospital of Anhui Medical University, 218 Jixi Road, Shushan District, Hefei, 230022 Anhui China; 2 Anhui Medical University, Shushan District, Hefei, 230001 Anhui China

**Keywords:** Gastric cancer, miR-140-3p, SNHG12, RNA-binding protein HuR, FAM38B, Proliferation, Nuclear transportation, Metastasis, mRNA stability

## Abstract

**Background:**

Gastric cancer (GC) is a malignant tumor and microRNAs (miRNAs) are closely connected to GC development. The purpose of this study is to investigate the effect of miR-140-3p on the occurrence and metastasis of GC.

**Methods:**

We detected miR-140-3p expression in GC cells and tissues. The correlation between miR-140-3p and prognosis and clinicopathological features in GC was analyzed. The role of miR-140-3p in GC cell migration, invasion, and proliferation was analyzed. The model of tumor transplantation and metastasis in nude mice was established, and the effect of miR-140-3p on the development and metastasis of GC was assessed. The relation between miR-140-3p and SNHG12 and the relations among HuR, SNHG12, and FAM83B were analyzed.

**Results:**

miR-140-3p was poorly expressed in GC. GC patients with low miR-140-3p expression had a poor prognosis and unfavorable clinicopathologic features. Overexpression of miR-140-3p inhibited GC cell migration, invasion, and proliferation, and inhibited the development and metastasis of GC. miR-140-3p directly bound to SNHG12 in GC tissues and downregulated SNHG12 expression. SNHG12 overexpression induced HuR nuclear transportation. HuR can bind to FAM83B and up-regulate the mRNA level of FAM83B. Overexpression of SNHG12 or FAM83B reduced the inhibition of overexpression of miR-140-3p on GC.

**Conclusion:**

miR-140-3p directly bound to SNHG12 in GC and down-regulated the expression of SNHG12, reduced the binding of SNHG12 and HuR, thus inhibiting the nuclear transportation of HuR and the binding of HuR and FAM83B, and reducing the transcription of FAM83B, and finally inhibiting the growth and metastasis of GC.

## Introduction

Gastric cancer (GC) represents a major public health issue as the fourth most common cancer and the second major cause of cancer-related death worldwide [1]. However, due to the asymptomatic nature, GC is often diagnosed in the late stage, at which point there are limited treatment options [2]. Currently, surgery is regarded as the only radical treatment [1]. Both postoperative recurrence and distant metastasis are thorny problems [3]. Therefore, it is essential to study the pathogenesis of GC and search for more effective treatment methods.

MicroRNAs (miRNAs) play a key role in gene expression and control both physiological and pathological processes. They are also crucial in the occurrence and progression of various diseases [4]. It has been verified that miRNAs can modulate the occurrence and metastasis of GC [5]. It has been identified that miR-140-3p inhibits the progression and metastasis in various cancers [6]. Results of previous studies have demonstrated that miR-140-3p is poorly expressed in GC [7, 8]. Nevertheless, the exact role of miR-140-3p in GC remains unknown and further investigation is necessary.

Long noncoding RNAs (lncRNAs) have been identified to promote the development, metastasis, and drug resistance of cancer cells [9]. MiRNAs can directly bind to lncRNAs to regulate the stability of lncRNAs, thereby regulating the expression level of lncRNAs [10, 11]. In the current study, the lncRNAs binding to miR-140-3p were predicted through the Starbase database. SNHG12 is commonly involved in many cancers in the contexts of tumorigenesis, migration, and drug resistance, including GC [12, 13]. It was found in a previous study that SNHG12 is highly expressed in GC [14]. Poor survival in GC patients can be predicted by SNHG12 which can be used as a biomarker [15]. Studies have shown that lncRNAs localized in the cytoplasm can bind to certain proteins in the cytoplasm, such as RNA-binding protein; then, they can regulate the activity and expression of the binding protein and affect the expression of the downstream gene in binding protein [16, 17]. Previous research has shown that SNHG12 can bind to RNA-binding protein Human antigen R (HuR) [18]. HUR post-transcriptionally modulates its target genes by stabilizing their mRNAs, and it is involved in cell growth and tumorigenesis in GC [19, 20]. In the progression of a wide range of human cancers, the family with sequence similarity 83 member B (FAM83B) has been proved to serve as an oncogene [21]. FAM83B expression can be stabilized by the combination of HuR with lncRNAs, thus promoting cell proliferation in GC [22]. Nevertheless, at home and abroad, the role and mechanism of miR-140-3p in the occurrence and metastasis of GC have not been reported. This study aims to explore the role of miR-140-3p in the occurrence and metastasis of GC, thus providing a new theory for the treatment of GC.

## Materials and methods

### Ethics statement

This study was authorized by the Ethical Committee of The First Affiliated Hospital of Anhui Medical University. All procedures were performed according to the Declaration of Helsinki. Animal experiments were conducted based on the minimized animal number and the least pains according to the Guide for the Care and Use of Laboratory Animals formulated by the National Institutes of Health [23].

### Collection of tissue specimens

GC tissues and matched adjacent non-tumoral tissues were collected from 60 GC patients (36 males and 24 females, aging from 38 to 77, with an average age of 63.25 years) admitted to The First Affiliated Hospital of Anhui Medical University. None patients had received any radiation and chemotherapy treatment. We have got informed consent from each patient. All patients were diagnosed by two experienced pathologists and the tumor stage was determined according to the TNM staging system of the American Joint Committee on Cancer (AJCC 7 Edition, 2010). The cancer tissues obtained by surgery were then immediately frozen in liquid nitrogen and stored at − 80 ℃ environment. Table 2 shows the clinicopathological features of patients, including sex, age, tumor size, TNM stage, and lymphatic metastasis.

### Cell culture

Human GC cell lines AGS, HS-746T, HGC27, MKN45, and NCIN87s and immortalized gastric mucosa GES-1 cells were bought from the cell bank of the Chinese Academy of Sciences (Shanghai, China). Cells were cultured in Dulbecco's modified Eagle medium (DMEM) (Gibco, BRL, San Francisco, USA) containing 10% fetal bovine serum (FBS, HyClone, Carlsbad, CA, USA), 100 μg/mL of streptomycin, and 100 U/mL penicillin in a humid environment at 37 ℃ and 5% CO_2_.

### Cell treatment

miR-140-3p inhibitor and its negative control (GenePharma, Shanghai, China) were transfected into MKN45 cells using Lipofectamine 2000 (Invitrogen). FAM83B pcDNA, HuR pcDNA, and their negative controls (GenePharma) were transfected into AGS cells using Lipofectamine 2000 (Invitrogen). Lentiviral shRNA targeting SNHG12 (sh-SnHG12-1, sh-SnHG12-2, sh-SnHG12-3) and its negative control (sh-NC) (OBiO, Shanghai, China) were transfected into AGS cells. Stable cell lines were obtained by puromycin screening.

### Cell counting kit-8 (CCK-8) assay

AGS and MKN45 cells were re-suspended in the DMEM and seeded on 96-well plates with 5000 cells and 200 μL per well. CCK-8 (10 μL) was added to each well after culture at 37℃ for 0 h, 24 h, 48 h, and 72 h, respectively. After culture at the same condition for 2 h, the absorbance was measured at 450-nm with a microplate reader.

### Colony formation assay

AGS and MKN45 cells at a density of 1000 cells/well were seeded into 6-well plates three times. After 10 days, cells were gently rinsed with phosphate-buffered saline (PBS) 3 times, fixed by 4% paraformaldehyde, and stained by 0.1% crystal violet (Sigma). The number of colonies was counted using an optical microscope (Olympus, Japan).

### Transwell assays

Transfected AGS and MKN45 cells were incubated in 24-well plates with an 8-mm pore size polycarbonate membrane (Corning, New York, USA) for the migration assay. To conduct the invasion test, cells in the serum-free medium were put into the apical chamber coated with Matrigel (Sigma-Aldrich), and the medium containing 1% FBS was added to the basolateral chamber. After 24 h, the cells in the apical chamber were wiped using cotton swabs. Subsequently, cells on the surface of the lower membrane were fixed by 4% paraformaldehyde and stained by 0.1% crystal violet. The cells in five random visual fields were counted under an inverted microscope (Laika, Germany).

### RNA stability assay

Actinomycin D (5 µg/mL) was used to treat AGS cells. The cells were collected after culture for 0 h, 3 h, 6 h, and 9 h. The Trizol reagent was used to extract the RNA. The levels of SNHG12 and FAM83B were measured by Reverse Transcription-Quantitative Polymerase Chain Reaction (RT-qPCR).

### Immunofluorescence

The slides were put into 24-well plates, washed with PBS 3 times, then fixed with 4% paraformaldehyde for 15 min, and then treated with 0.5% Triton X-100. The cells were sealed with 10% goat serum for 10 min at room temperature after washing with PBS. Cells were cultured overnight at 4 ℃ with primary antibody (ab200342, Abcam, Shanghai, China). Next, cells were cultured with goat anti-rabbit IgG H&L (Alexa Fluor® 488) (AB150077, Abcam) at 37 ℃ for 1 h in the dark. The nuclei were stained with 4,6-diamino-2-phenyl indole (DAPI) and incubated for 5 min at room temperature in the dark. Liquids with anti-fluorescence quenching were used to seal the slides. Images were captured using a fluorescence microscope (Olympus).

### Subcellular fractionation assay

The Paris kit (Life Technologies, New York, USA) was used to separate the nuclear and cytoplasmic components of AGS and MKN45 cells. Then, the cells were treated with cytoplasmic protein extractant A and B after washing with PBS. The supernatant was centrifuged at 12,000*g* at 4 ℃ for 10 min to separate the nucleus and cytoplasm. The nucleus was centrifuged at 12,000*g* after resuspending at 4 ℃ for 10 min. The supernatant was collected as a nuclear extract for the subsequent analysis.

### RNA fluorescence in situ hybridization (FISH)

The FISH kit (RiboBio Co., Ltd, Guangzhou, China) was used to detect the subcellular localization of SNHG12 in AGS cells and MKN45 cells. In brief, GC cells were fixed by 4% paraformaldehyde. After permeabilization in PBS containing 0.5% Triton X-100, the cells were cultured with cy3-labeled specific probes SNHG12 (GenePharma). The cells were stained using DAPI. Images were obtained using the microscope (Olympus, Japan).

### RNA–protein immunoprecipitation (RIP)

EZ-Magna RIP kit (Millipore, Billerica, MA, USA) was applied to perform the RIP assay. AGS and MKN45 cells at 80–90% confluence were collected and then lysed by using the RIP lysis buffer. Cell extract of 100 μL was cultured by RIP buffer which contains magnetic beads conjugated with HuR (ab200342, Abcam) or Ago2 (ab186733, Abcam) or IgG (ab172730, Abcam) antibody at 4 ℃ for 6 h. Then the beads were rinsed by washing buffer. Then the compound was incubated with 0.1% SDS/0.5 mg/mL protease K (at 55 ℃ for 30 min) to remove the protein. A Nanodrop spectrophotometer (Thermo Scientific) was used to measure the RNA concentration. RNA quality was assessed using a biological analyzer (Agilent, Santa Clara, CA, USA). At last, the immunoprecipitated RNA was analyzed by RT-qPCR.

### Dual-luciferase assay

The 3'-UTR of SNHG12 containing the binding site of miR-140-3p was put into the pMIR-REPORT plasmid (Thermo Fisher Scientific, MA, USA) to construct wild-type plasmid (SNHG12 WT). The 3'-UTR of SNHG12 containing mutant sequences was put into the pMIR-REPORT plasmid to construct the SNHG12 mutant type (SNHG12 WUT). AGS and MKN45 cells were transfected with miR-140-3p mimic or mimic NC (GenePharma) using Lipofectamine 2000 (Invitrogen). Cells were lysed 48 days after transfection. Luciferase activity was measured using a dual-luciferase reporter assay system (Promega, Madison, Wisconsin).

### Xenograft tumors in nude mice

Five-week-old male BALB/C nude mice were provided by Vital River Company (License No. SYXK (Beijing) 2017-0033). AGS cells with stable overexpression of miR-140 or joint overexpression of SNHG12 were collected. They were resuspended in PBS on ice. Then, AGS cells (4 × 10^6^/150 μL) were injected into the right thigh of each mouse. From the 7th day, the tumor volume (Volume = length × width^2^) was examined every 3 days. Mice were euthanized with 100 mg/kg of sodium pentobarbital in the abdominal cavity 21 days after injection. In each group, the tumors of 6 mice were removed for immunohistochemistry and the tumors of the remaining 6 mice were used for RT-qPCR.

### Metastatic model of GC

AGS cells with stable overexpression of miR-140-3p or joint overexpression of SNHG12 were collected. After infection with luciferase reporter lentivirus, the cells were suspended to 2 × 10^7^/mL in icy PBS. Then, 100 μL of suspension cells were injected into the tail vein of the mice. Lung metastases were measured using bioluminescence imaging at the 3rd, 5th, and 7th weeks. D-luciferin sodium stock solution was prepared with 15 mg/mL PBS, and 150 mg/kg luciferin stock solution was intraperitoneally injected into the mouse to induce bioluminescence. All mice were immediately anesthetized with 2% isoflurane and imaged 10 min later. The intensity and position of bioluminescence in mice were detected using the biometer for living small animals (Caliper Life Sciences, USA). At last, the mice were sacrificed and their lungs were removed for Hematoxylin and eosin (HE) staining.

### Immunohistochemistry assay

Tumor tissues were blocked with goat serum for 20 min at room temperature after dewaxing, dehydration, and antigen repair. Then, goat serum was removed. Tissue slices were cultured with primary bodies anti-Ki67 (ab16667, Abcam) and HuR (ab200342, Abcam) overnight at 4 ℃, then, were incubated with secondary antibody (ab205718, Abcam). Diaminobenzidine (DAB) complex (Zhongshan Jinqiao, Beijing, China) was used as the chromogen. The 15% hematoxylin was used to counterstain the nuclei. Images were taken using microscopes.

### Hematoxylin and eosin (HE) staining

Paraffined slices of lung tissue were dewaxed with xylene and ethanol. The tissue slices were stained with hematoxylin for 10 min and then rinsed with water to remove residual color. Then, slices were differentiated for several seconds and rinsed with water. Fifteen minutes after slices turned blue, slices were differentiated with 95% ethanol and stained with alcoholic eosin for 30 s. After being dewaxed with gradient hexanol, the slices were put into xylene carbolate (Sinopharm Chemical Reagents Co., Ltd., Shanghai, China) and sealed with neutral gum. At last, the metastatic lesion of the lung tumor was observed under a microscope.

### Reverse Transcription-Quantitative Polymerase Chain Reaction (RT-qPCR)

TRIzol reagent (Invitrogen) was used to extract all the RNA, which was then reversed transcribed into cDNA using a reverse transcription kit (Takara, Dalian, China). Primers are exhibited in Table 1. GAPDH was applied as an internal reference. The relative expression was calculated based on the 2−ΔΔ CT method [24].

**Table 1:** PCR primers sequences

Species	Sequences(5′-3′)
SNHG12	F: ATGGTGGTGAATGTGGCACG
	R: GCACAGCTCCAGAAACAAGC
miR-140-3p	F: CCTGGTTACCACAGGGTAGA
	R: TCAACTGGTGTCGTGGAGTC
FAM83B	F: ATGGAGACCTCATCAATGCT
	R: GTTGATATGAGCGATAAACACC
U6	F: TCGCTTCGGCAGCACATATACT
	R: GCTTCACGAATTTGCGTGTCATC
GAPDH	F: ATGGTTTACATGTTCCAATATGA
	R: TTACTCCTTGGAGGCCATGTGG

### Western blot

The radioimmunoprecipitation (RIPA) buffer (SolarBio, Beijing, China) containing protease inhibitors and phosphatase inhibitors was used to extract the protein from tissues and cells. The supernatant of the cell extract was isolated on 10% SDS-PAGE gel. Then it was transferred to polyvinylidene fluoride (PVDF) membrane which was then sealed in 5% bovine serum albumin (BSA) for 1 h. The membrane was cultured with the antibodies against HuR (ab200342, 1:1000, Abcam) and β-actin (ab8227, 1:1000, Abcam) at 4 ℃ overnight. TBST (SolarBio, China) was used to rinse the stain on the membrane three times. Then the membrane was cultured with the secondary antibody at room temperature for 2 h. NIH Image J (National Institutes of Health, Bethesda, Maryland, USA). GAPDH was utilized to evaluate gray value as the internal reference.

### Bioinformatics analysis

The expression of miR-140-3p, SNHG12, and FAM83B in GC was predicted using the Starbase (http://starbase.sysu.edu.cn/index.php) [25] and TCGA (http://ualcan.path.uab.edu/analysis.html) [26]. The expression of FAM83B in GC was predicted using the GEPIA (http://gepia.cancer-pku.cn/) [27]. The expression of miR-140-3p and SNHG12 in GC was analyzed using the Kaplan–Meier Plotter (http://kmplot.com/analysis/index.php?p=service&cancer=liver_rnaseq) [28]. The lncRNAs binding to miR-140-3p were predicted using the Starbase (http://starbase.sysu.edu.cn/index.php). The binding score of SNHG12 and HUR and that of HUR and FAM83B were predicted using the RNA–Protein Interaction Prediction (RPISeq) (http://pridb.gdcb.iastate.edu/RPISeq/) [29].

### Statistical analysis

SPSS21.0 (IBM Corp. Armonk, NY, USA) and GraphPad Prism 8.0 (GraphPad Software Inc., San Diego, CA, USA) were used for statistical analysis and plotting. Tests for normality and homogeneity of variance were conducted, which verified the normal distribution and homogeneity of variance. Comparisons between two groups were conducted using the t-test. Comparisons among various groups were conducted using one-way analysis of variance (ANOVA) or two-way ANOVA, followed by Tukey's Multiple Verbs Test or Sidak's multiple comparisons test. Counting data were represented by the number of cases. The comparisons among data in panels were conducted using Fisher. The relation between miR-140-3p and prognosis and clinicopathologic features of GC patients was analyzed using Kaplan–Meier survival curves and log-rank. The correlation between miR-140-3p and SNHG12, SNHG12, and FAM83B was analyzed using Pearson correlation analysis. *P* value was procured from a bilateral test. *P* < 0.05 stated that the difference had statistical significance. *P* < 0.01 indicated that the difference was highly statistically significant.

## Results

### miR-140-3p was poorly expressed in GC cells and tissues and was correlated with prognosis and clinicopathologic features of GC patients

**Fig. 1:**
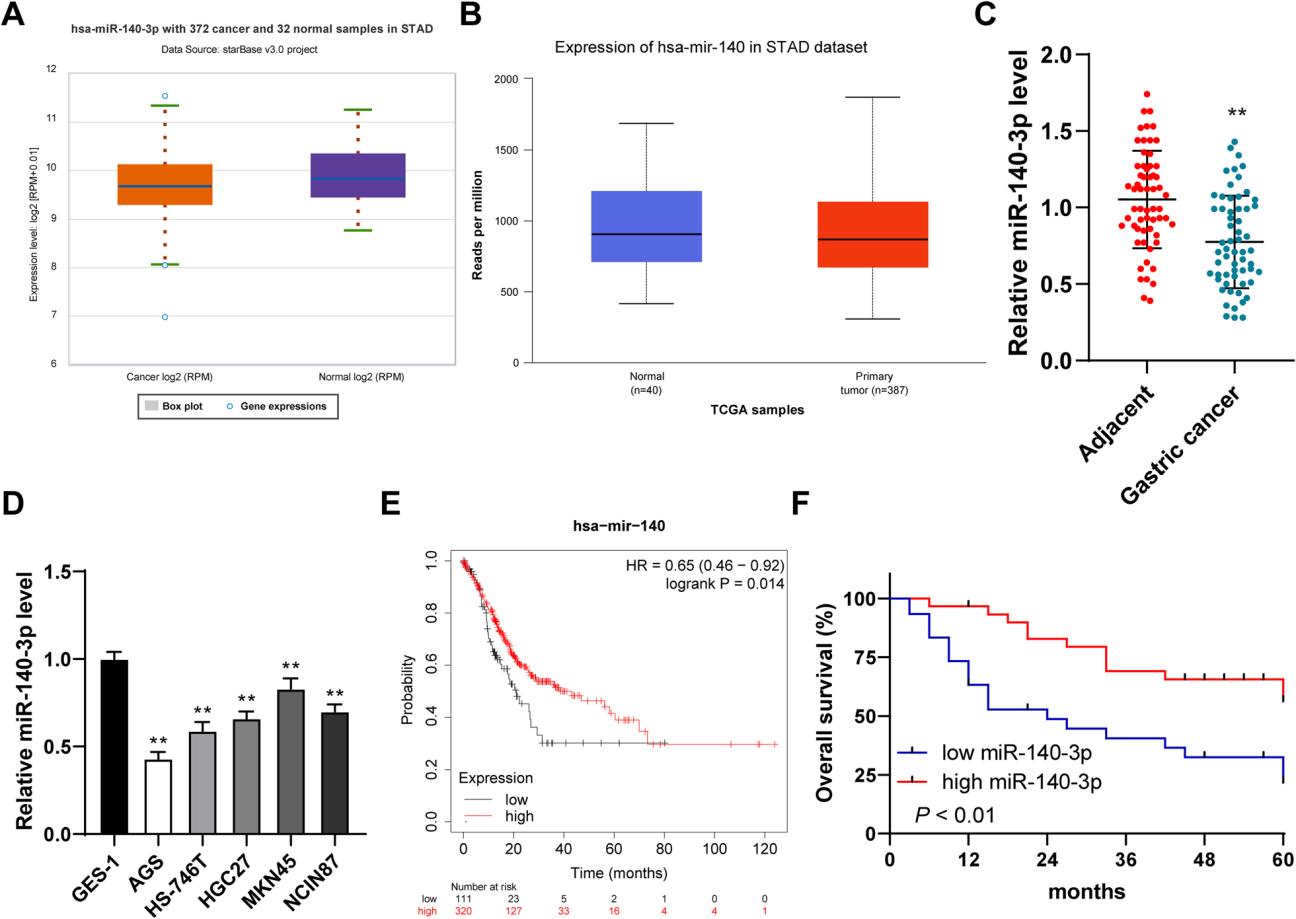
miR-140-3p was poorly expressed in tissues and cells of GC and was related to the prognosis and clinicopathologic features of patients with GC. **A**, **B** The expression of miR-140-3p in gastric adenocarcinoma was predicted by the Starbase and UALCAN database. **C** The expression of miR-140-3p in GC tissues and adjacent tissues was detected using RT-qPCR. **D** The expression of miR-140-3p in GC cell lines was detected using RT-qPCR. **E** The relation between miR-140-3p and the prognosis in patients with GC was analyzed with Kaplan–Meier Plotter. **F** The relation between the expression of miR-140-3p in GC tissues and prognosis in patients was analyzed by Kaplan–Meier. N = 60. The cell experiment was repeated 3 times independently. Data in **D** were presented as mean ± standard deviation. Comparisons between two panels in **C** were analyzed using the t-test. Comparisons of data in **F** were performed using the Log Rank test. Comparisons of data in **D** were performed using one-way ANOVA, followed by Tukey's multiple comparisons test. ^**^p < 0.01

miRNAs regulate the occurrence and metastasis of GC [30–32]. It has been confirmed in a previous study that miR-140-3p is poorly expressed in GC [8]. However, its regulatory mechanisms on the occurrence and metastasis of GC remain unknown. Firstly, it was predicted that miR-140-3p has poor expression in gastric adenocarcinoma cells through the data from the Starbase and TCGA (Fig. 1A, B). In addition, RT-qPCR detected that miR-140-3p was poorly expressed in GC tissues (*P* < 0.01, Fig. 1C). RT-qPCR detected that miR-140-3p was poorly expressed in GC cells (*P* < 0.01, Fig. 1D). Sixty patients with GC were assigned to the group of high expression and the group of low expression based on the median of miR-140-3p in GC tissues [20] to analyze the correlation between the miR-140-3p expression and clinicopathological features in these 60 patients with GC. We found that the miR-140-3p expression was correlated with tumor size, lymph node metastasis degree, and TNM stage (*P* < 0.05, Table 2). Kaplan–Meier Plotter database was used to predict the relation between the expression of miR-140-3p and prognosis and clinicopathologic features of GC patients. The survival time of patients with low miR-140-3p expression was shorter than that of patients with high miR-140-3p expression (Fig. 1E). Then, 60 patients with GC were subjected to the Kaplan–Meier survival analysis. We found that the miR-140-3p expression was related to the prognosis of GC patients and GC patients with low expression of miR-140-3p had shorter overall survival (*P* < 0.01, Fig. 1F). In short, miR-140-3p was poorly expressed in GC and was related to the prognosis and clinicopathologic features of GC patients.

**Table 2:** Correlation between miR-140-3p expression and clinicopathological characteristics of gastric cancer patients

Characteristic	Number	Low expression (N = 30)	High expression (N = 30)	*P* value
Gender				0.292
Male	36	16	20	
Female	24	14	10	
Age				0.602
≤ 65	26	14	12	
> 65	34	16	18	
Histologic differentiation				0.301
Well, moderate	32	18	14	
Poor	28	12	16	
Tumor size				0.035
≤ 5 cm	24	8	16	
> 5 cm	36	22	14	
Lymph node metastasis				0.003
Negative	21	5	16	
Positive	39	25	14	
TNM stage				0.009
I, II	26	8	18	
III, IV	34	22	12	
Tumor site				0.432
Antrum	35	19	16	
Cardia	25	11	14	

### miR-140-3p overexpression inhibited the migration, invasion, and proliferation of GC cells

**Fig. 2:**
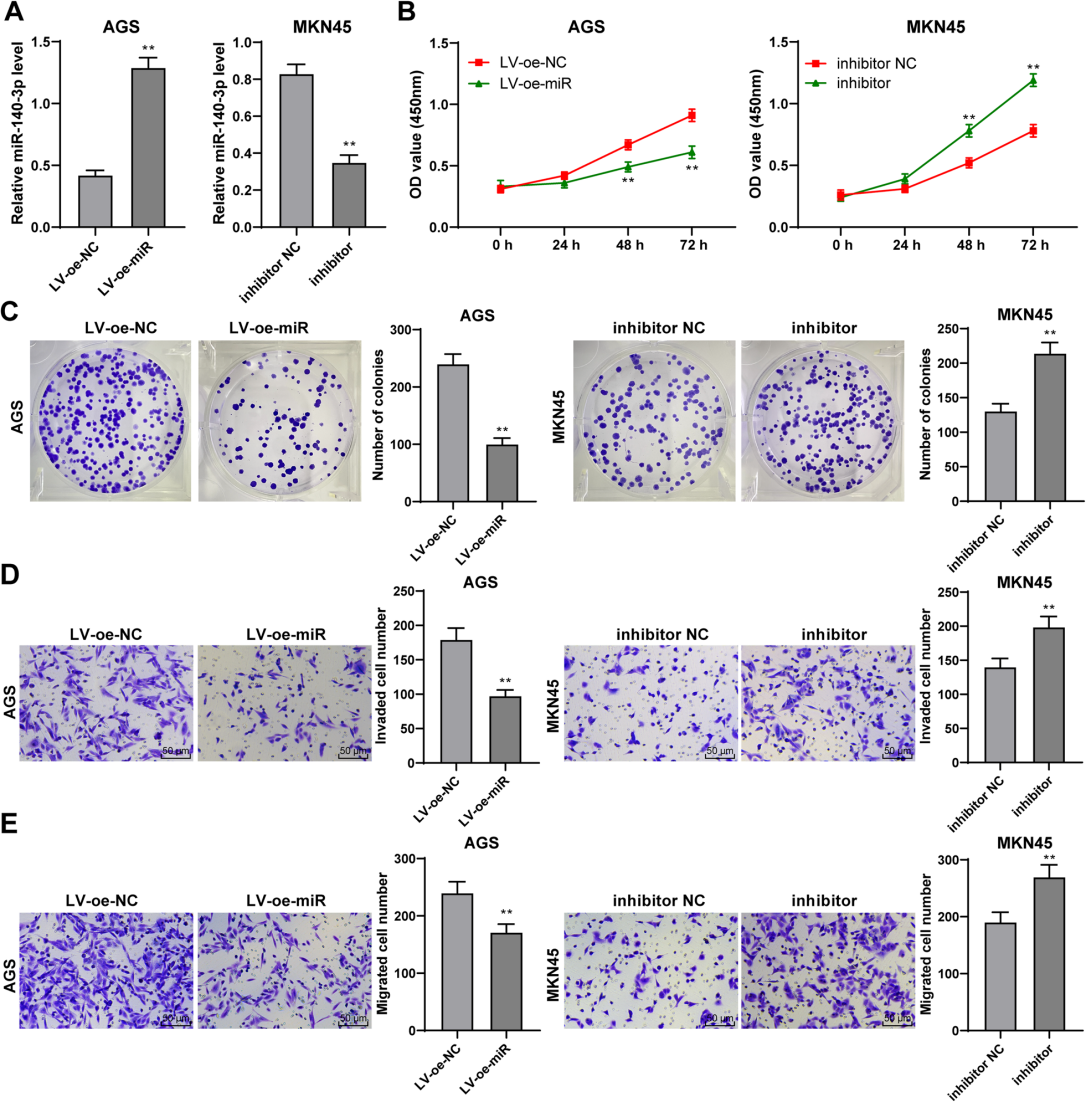
Overexpression of miR-140-3p inhibited the proliferation, invasion, and migration of GC cells. miR-140-3p lentivirus overexpression vector was transfected into AGS cells with low expression. miR-140-3p inhibitor was transfected into MKN45 cells with high expression. **A** The expression of miR-140-3p in GC cells was detected using RT-qPCR. The proliferation of cells was detected by CCK-8 assay (**B**) and clone formation assay (**C**). **D**, **E** The invasion and migration of cells were detected by Transwell assays. The experiment was repeated3 times independently. Data were presented as mean ± standard deviation. Comparisons among panels in **A**, **C**, **D**, **E** were performed using the t-test. Comparison of data in B was performed using two-way ANOVA, followed by Tukey's multiple comparisons test. **p < 0.01. LV-oe-miR: The lentiviral overexpression vector of miR-140-3p; LV-oe-NC: negative control of lentiviral overexpression vector; inhibitor: miR-140-3p inhibitor

Then, miR-140-3p lentivirus overexpression vectors were used to infect AGS cells with relatively poor miR-140-3p expression to investigate the role of miR-140-3p in GC cells. miR-140-3p expression in AGS cells was up-regulated (*P* < 0.01, Fig. 2A). The miR-140-3p inhibitor was transfected to MKN45 cells with relatively high miR-140-3p expression, and then, the expression of miR-140-3p in cells was reduced (*P* < 0.01, Fig. 2A). It was founded that the proliferation of AGS cells was reduced after overexpression of miR-140-3p but increased after downregulation of miR-140-3p (*P* < 0.01, Fig. 2B, C). Moreover, miR-140-3p overexpression inhibited the invasion and migration of AGS cells. Downregulation of miR-140-3p promoted the invasion and migration of MKN45 cells (*P* < 0.01, Fig. 2D, E). All in all, overexpression of miR-140-3p inhibited the migration, invasion, and proliferation of GC cells.

### Overexpression of miR-140-3p inhibited the development and metastasis of GC

**Fig. 3:**
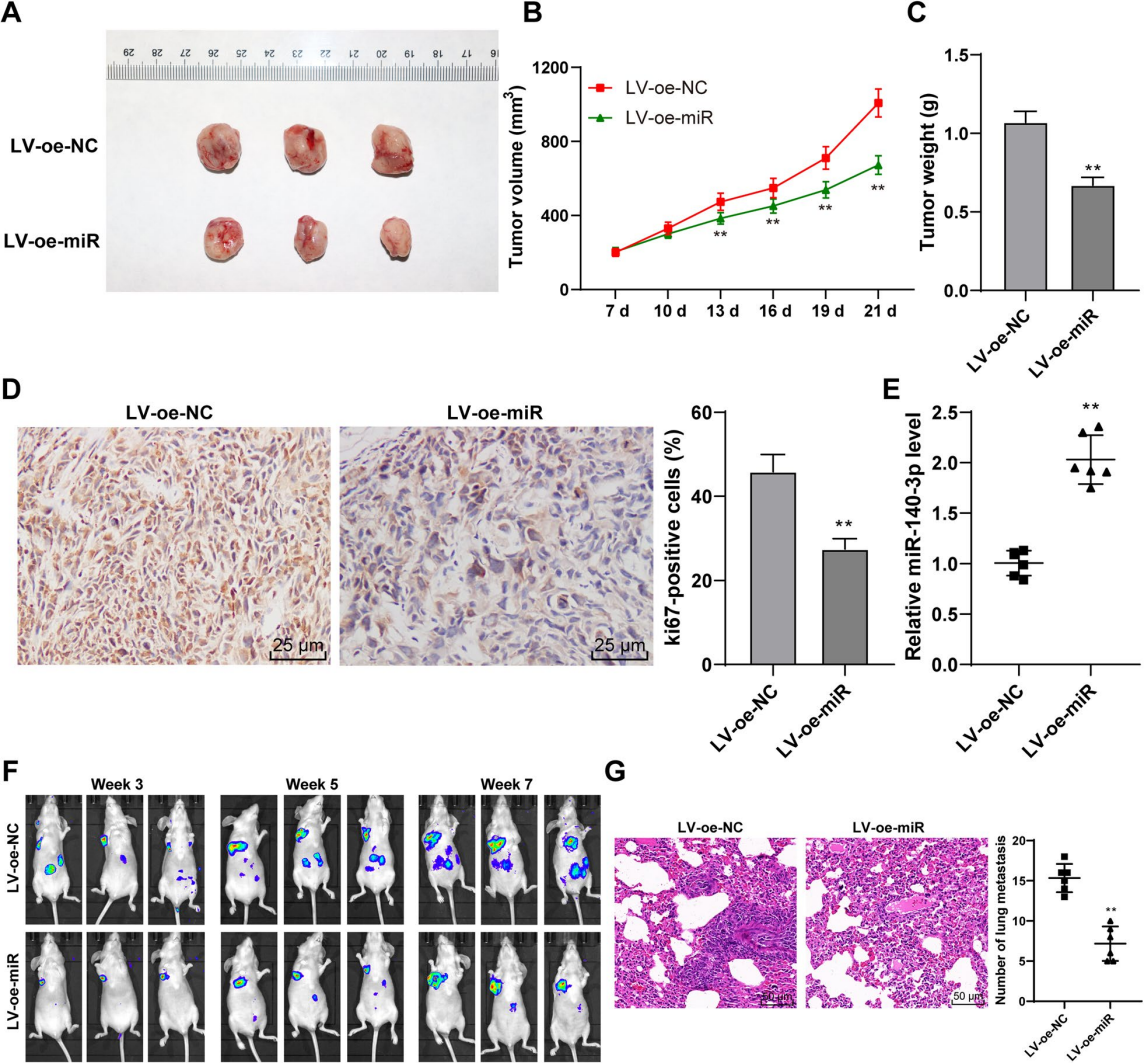
Overexpression of miR-140-3p inhibited the growth and metastasis of GC. Nude-mouse transplanted tumor model was established using AGS cells with overexpression of miR-140-3p. **A** Representative image of a transplanted tumor model. **B** The volume of the tumor during modeling. **C** The weight of the tumor on day 21. **D** The positive rate of ki67 in GC tissues was measured by immunocytochemistry. **E** The expression of miR-140-3p in tumor tissues was detected by RT-qPCR. Nude-mice lung metastasis models were established using AGS cells with overexpression of miR-140-3p. **F** Metastatic area of GC was detected using an in vivo imaging system. **G** The number of pulmonary metastasis was observed by HE staining. Data in **B**, **C**, **D** were presented as mean ± standard deviation. Comparison among panels in **C**, **D**, **F**, **G** was performed using one-way ANOVA. Comparison of data in B was performed using two-way ANOVA, followed by Tukey's multiple comparisons test. **p < 0.01. LV-oe-miR: The lentiviral overexpression vector of miR-140-3p. LV-oe-NC: negative control of lentiviral overexpression vector

Then, AGS cells with overexpression of miR-140-3p were used to establish the nude mouse transplanted tumor models (Fig. 3A). The results showed that tumor growth was inhibited (*P* < 0.01, Fig. 3B), and tumor weight was significantly reduced (*P* < 0.01, Fig. 3C) after overexpression of miR-140-3p. Ki67 is the marker of proliferation [33]. Immunocytochemistry results exhibited that the positive expression rate of Ki67 protein in tumor tissues was reduced by the overexpression of miR-140-3p (*P* < 0.01, Fig. 3D). miR-140-3p expression in tumor tissues was markedly increased after the injection of AGS cells (*P* < 0.01, Fig. 3E). In addition, the lung metastatic model was established by injecting AGS cells with overexpression of miR-140-3p into THE caudal vein. The metastases were observed in vivo using an in vivo imaging system. It was observed that miR-140-3p overexpression could inhibit tumor metastasis (Fig. 3F). The results of HE staining showed that after overexpression of miR-140-3p, the number of lung metastases was also significantly reduced. In conclusion, overexpression of miR-140-3p inhibited the development and metastasis of GC.

### miR-140-3p directly bound to SNHG12 in GC tissues and reduced its stability

**Fig. 4:**
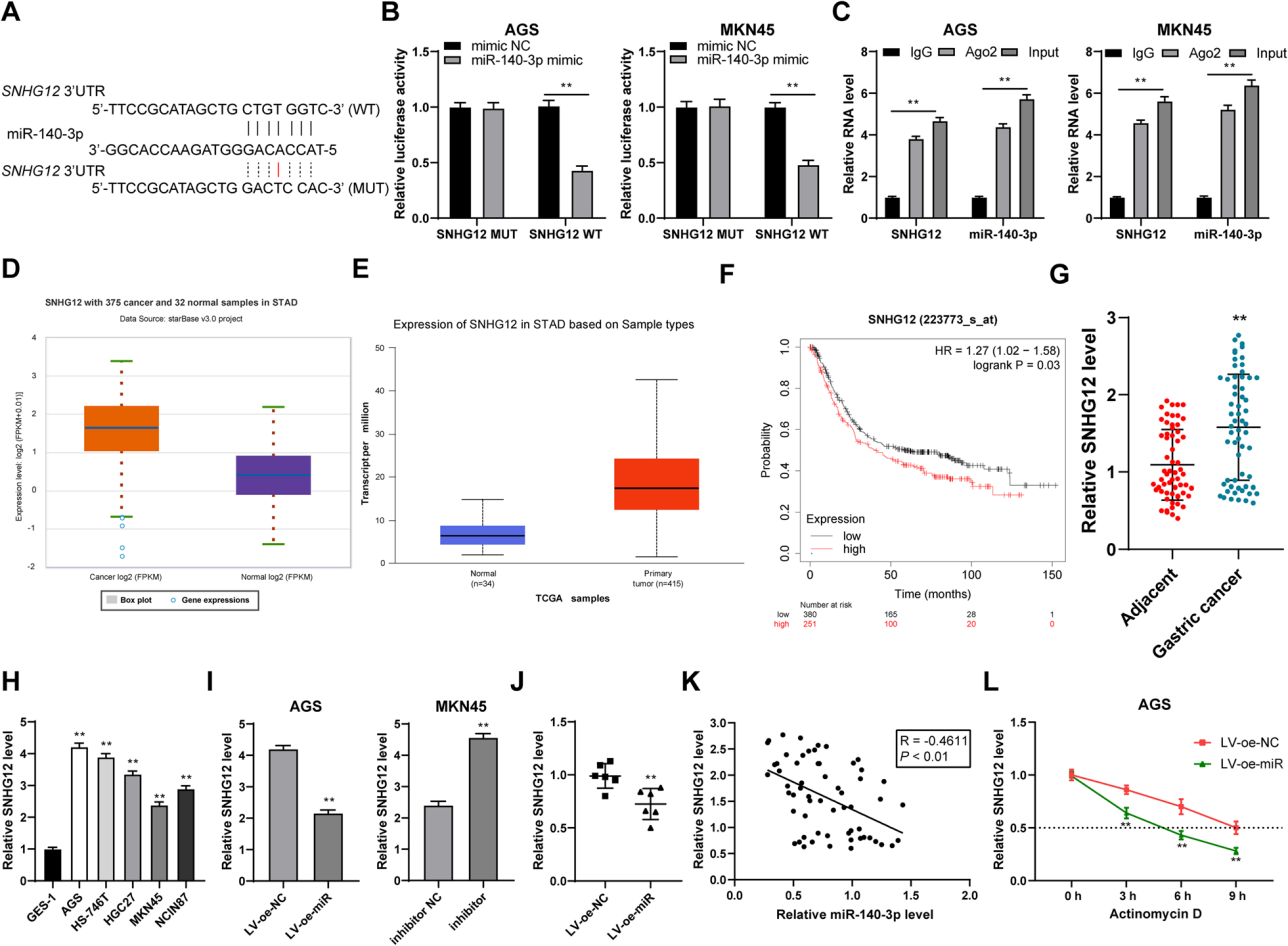
miR -140-3p bound to lncRNA snHG12 in GC tissues and reduced its stability. **A** Binding site of miR-140-3p and SNHG12 predicted using the Starbase database. **B**–**C** The binding relationship of 140-3p and SNHG12 in GC cells was verified using dual luciferase assay and RIP. **D**–**E** Expression of SNHG12 in gastric adenocarcinoma was predicted using the Starbase and UALCAN databases. **F** The relation between SNHG12 and the prognosis of GC patients was analyzed using Kaplan–Meier Plotter database. **G** The expression of SNHG12 in GC tissues and adjacent tissues was detected using RT-qPCR. **H**, **I** The expression of SNHG12 in GC cells was detected using RT-qPCR, N = 6. **J** The expression of SNHG12 in transplanted tumor tissues was detected using RT-qPCR. **K** Relevance between miR-140-3p and SNHG12 was analyzed by Pearson correlation analysis. **L** After GC cells with low expression of miR-140-3p were treated with actinomycin **D**, the half-life period of SNHG12 was detected using RT-qPCR. The cell experiment was repeated 3 times independently. Data in **B**, **C**, **H**, **K** were presented as mean ± standard deviation. Comparison between two groups in panels **G**, **I**, and **J** was performed using the t-test. Comparison of data in H was analyzed by using one-way ANOVA and comparison of data in **B**, **C**, **L** was analyzed by using two-way ANOVA, followed by Tukey's multiple comparisons test or Sidak's multiple comparisons test. **p < 0.01. LV-oe-miR: The lentiviral overexpression vector of miR-140-3p; LV-oe-NC: negative control of lentiviral overexpression vector; inhibitor: miR-140-3p inhibitor

Then, we further investigated the downstream mechanisms of miR-140-3p regulating the development and metastasis of GC. It has been reported that miRNA could directly bind to lncRNA to regulate the expression of lncRNA [10, 11]. The lncRNAs binding to miR-140-3p were predicted using the Starbase, among which, SNHG12 showed high expression in GC [9, 34]. The dual-luciferase assay was designed based on the binding sites of miR-140-3p and SNHG12 in the database (Fig. 4A), which exhibited that there was a binding relationship between miR-140-3p and SNHG12 in GC cells (*P* < 0.01, Fig. 4B). RIP experiment further confirmed their binding relationship (*P* < 0.01, Fig. 4C). The prediction results indicated that SNHG12 showed high expression in gastric adenocarcinoma cells (Fig. 4D, E) and the survival time of GC patients with highly-expressed SNHG12 was remarkably shorter than that of patients with poorly-expressed SNHG12 (Fig. 4F). The results of RT-qPCR exhibited that SNHG12 showed high expression in GC tissues and cells. The expression of SNHG12 in AGS cells with overexpression of miR-140-3p and the corresponding transplanted tumor tissue was reduced significantly. The expression of SNHG12 in MKN45 cells was increased significantly (*P* < 0.01, Fig. 4G–J). In GC tissues, miR-140-3p expression and SNHG12 were negatively correlated (*P* < 0.01, Fig. 4K). Then, GC cells with overexpression of miR-140-3p were treated with actinomycin D. It was exhibited that after the overexpression of miR-140-3p, the half-life period of SNHG12 was significantly shortened (*P* < 0.01, Fig. 4L). In short, miR-140-3p bound to SNHG12 in GC tissues directly and reduced SNHG12 stability.

### Overexpression of SNHG12 could reduce the inhibition of overexpression of miR-140-3p on the migration, invasion, and proliferation of GC cells

**Fig. 5:**
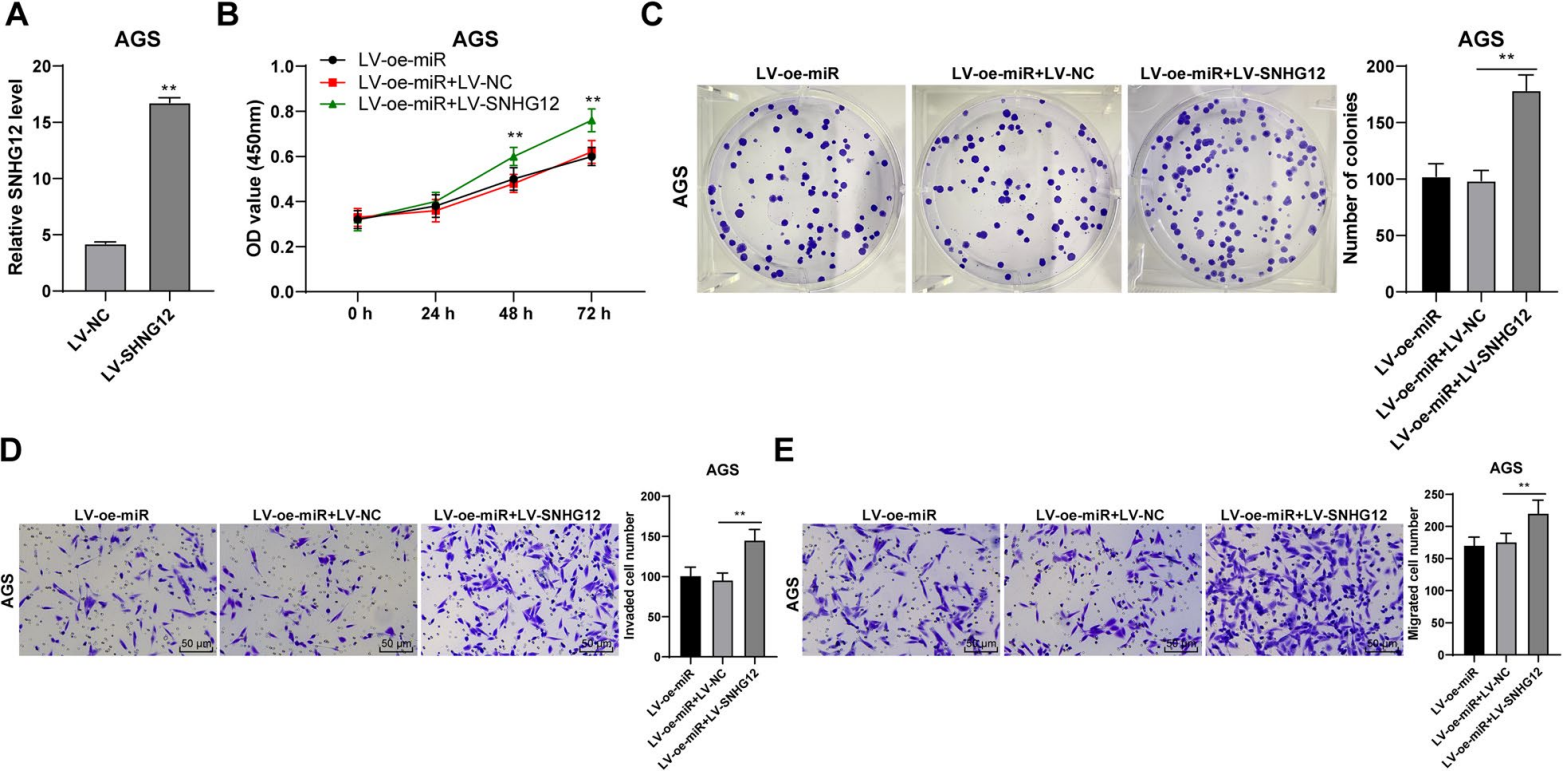
Overexpression of SNHG12 reduced the inhibition of overexpression of miR-140-3p on proliferation, invasion, and migration of GC cells. The lentiviral overexpression vector of SNHG12 was infected with AGS cells. **A** The expression of SNHG12 was detected using RT-qPCR. Joint intervention with lentiviral overexpression vector of miR-140-3p was performed. The proliferation of cells was detected using CCK-8 assay (**B**) and colony formation assay (**C**). **D**, **E** The invasion and migration of cells were detected by Transwell assays. The experiment was repeated three times independently. Data were presented as mean ± standard deviation. Comparison of data among panels in **A**, **C**, **D**, **E** was performed using the t-test. Comparison of data in B was performed using two-way ANOVA, followed by Tukey's multiple comparisons test. **p < 0.01. LV-oe-miR: The lentiviral overexpression vector of miR-140-3p; LV-SNHG12: The lentiviral overexpression vector of SNHG12; LV-NC: The lentiviral overexpression vector of NC

SNHG12 expression in AGS cells was up-regulated after AGS cells were infected with the lentivirus overexpressing vector of SNHG12 (*P* < 0.01, Fig. 5A). Then, the miR-140-3p lentivirus overexpression vector was used to treat the cells. It was found that compared with miR-140-3p overexpressing cells, the proliferation of cells with both overexpression of miR-140-3p and SNHG12 was significantly increased (*P* < 0.01, Fig. 5B, C), and the invaded and migrated cells were also increased (*P* < 0.01, Fig. 5D, E). Hence, it was demonstrated that SNHG12 could reduce the inhibition of miR-140-3p overexpression the migration, invasion, and proliferation of GC cells, and miR-140-3p inhibited the SNHG12 expression to regulate the migration, invasion, and proliferation of GC cells.

### SNHG12 bound to the RNA-binding protein HuR and induced HuR transporting from nuclei to cytoplasm

**Fig. 6:**
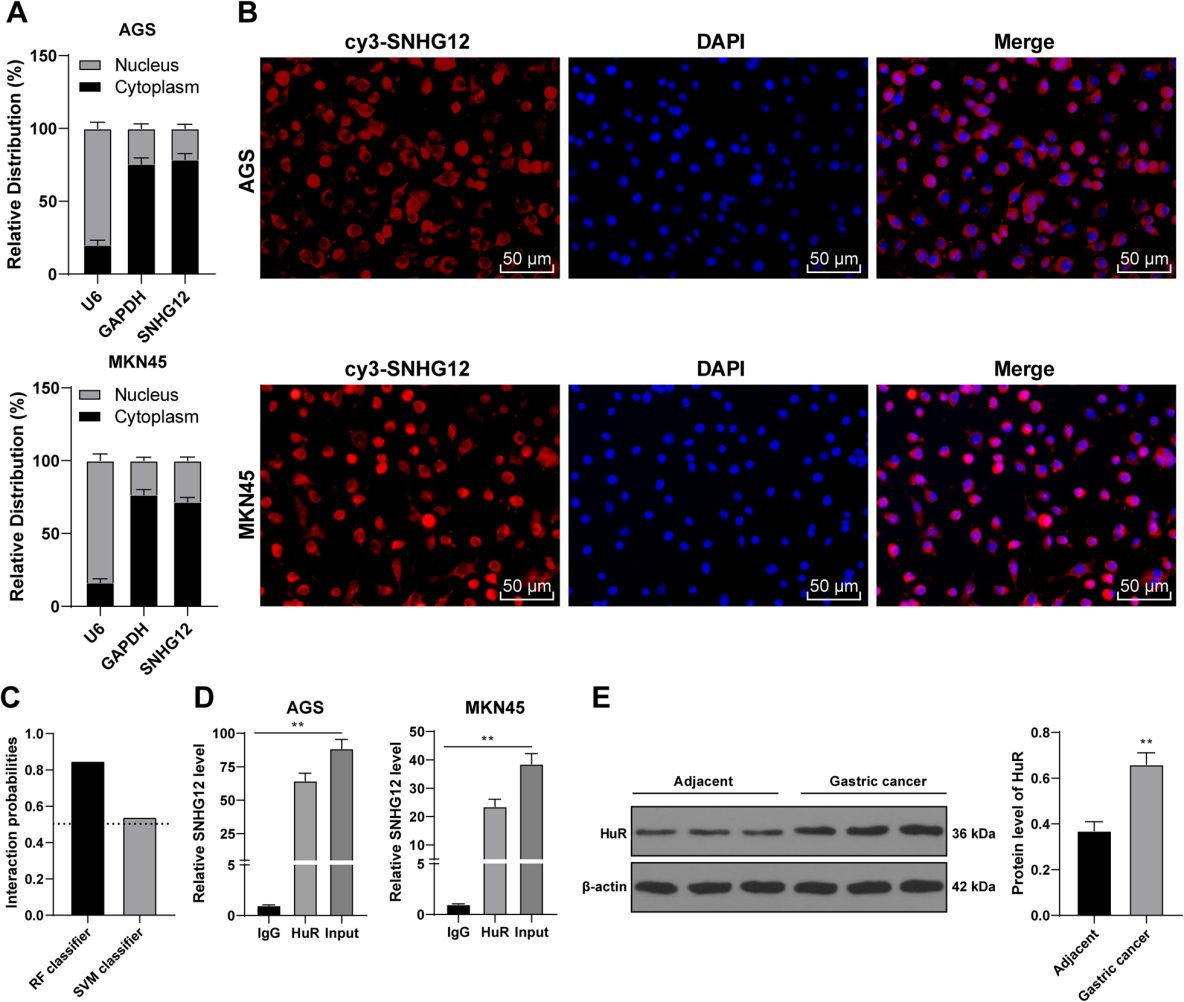

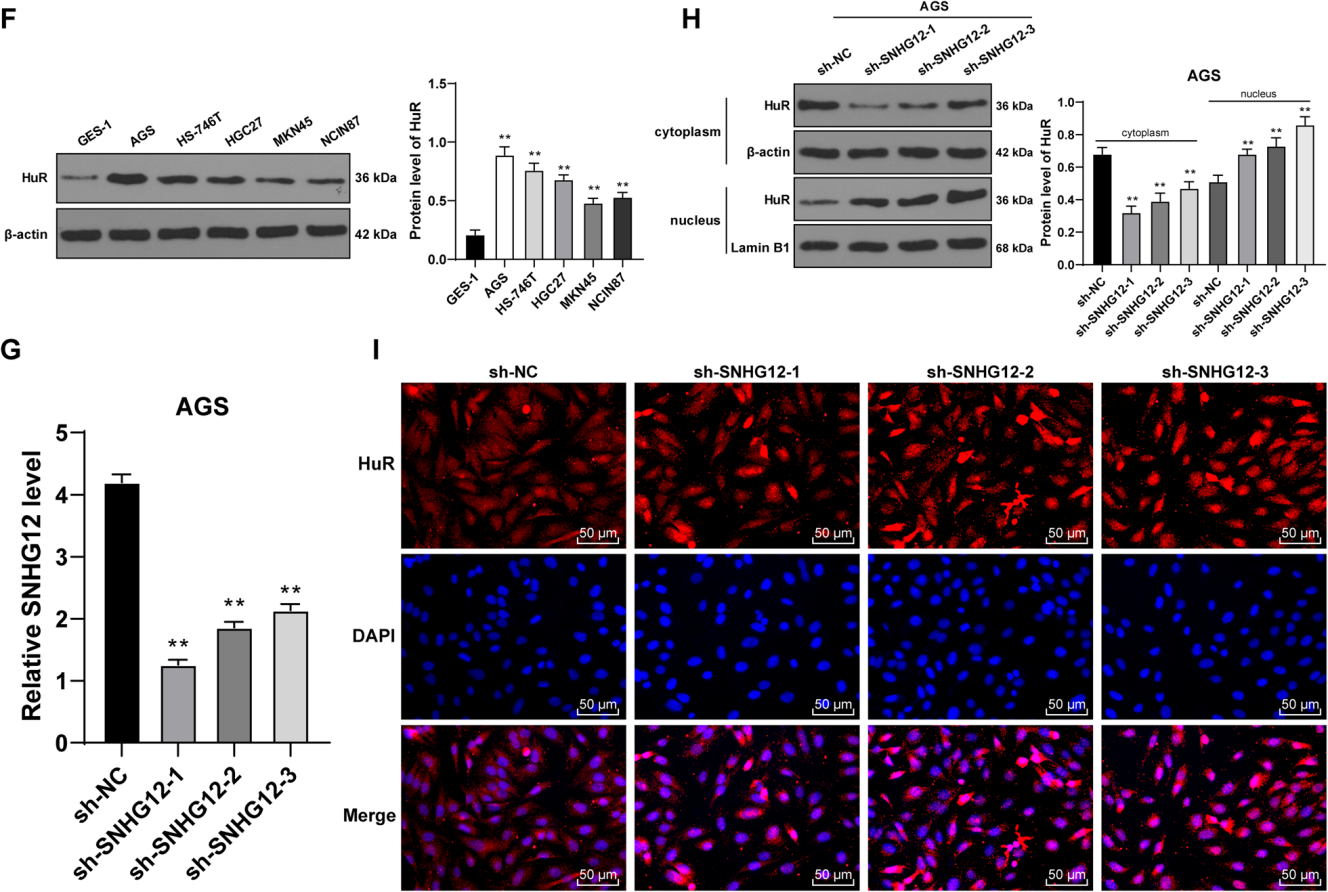
SNHG12 bound to RNA-binding protein HuR and induced transportation of HuR from the nuclei to the cytoplasm. **A**, **B** The location of SNHG12 was analyzed using subcellular fraction assay and RNA FISH; **C** The binding probability between SBHG12 and HuR was predicted using RNA–Protein Interaction Prediction (RPISeq) database. **D** The binding between SNHG12 and HuR was analyzed using RIP assay. **E**, **F** The protein expression of HuR in GC tissues and cells was detected by western blot. After the three designed SNHG12 shRNA were treated with AGS cells, NC shRNA was used as the control. **G** The expression in SNHG12 in AGS cells was detected by RT-qPCR. **H** The protein expression of HuR in GC cells was detected by western blot. **I** The aggregation of HuR in the cytoplasm was detected by immunofluorescence. The experiment was repeated three times independently. Data were presented as mean ± standard deviation. Comparison between data in panel **E** was analyzed by using the t-test. Comparison among groups in panels **D**, **F**, **G**, **H** was analyzed using one-way ANOVA, followed by Tukey's multiple comparisons test. *p < 0.05, **p < 0.01. sh-NC: NC shRNA; sh-SNHG12: SNHG12 shRNA

Then, we further explored the downstream mechanism of SNHG12. The location of SNHG12 in GC cells was detected using the subcellular fractionation assay and RNA FISH, which displayed that SNHG12 was chiefly located in the cytoplasm (Fig. 6A, B). In previous studies, it has been proved that lncRNA in the cytoplasm can bind to some kinds of protein such as RNA-binding protein, regulate the activity or expression of the binding protein, and affect the expression of downstream genes of the binding protein [16, 17]. SNHG12 can bind to HuR [18]. It was predicted that the binding probability of SNHG12 and HuR is very high (The scores of RF classifier and SVM classifier were 0.85 and 0.54 respectively.) (Fig. 6C). RIP assay verified that SNHG12 in GC cells was able to bind to HuR (*P* < 0.01, Fig. 6D). HuR showed high expression in GC tissues and cells (*P* < 0.01, Fig. 6E, F). Next, we transfected 3 pieces of shRNA of SNHG12 (sh-SNHG12) into AGS cells, and all of them could down-regulate the intracellular expression of SNHG12 (*P* < 0.01, Fig. 6G). After the knockdown of SNHG12, HuR expression in the cells was markedly decreased, while in the nucleus was increased (*P* < 0.01, Fig. 6H). The results of the immunofluorescence assay further verified that after SnHG12 knockdown, the aggregation of HuR in the cytoplasm was decreased (Fig. 6I). To sum up, SNHG12 bound to the RNA-binding protein HuR and induced the transportation of HuR from the nucleus to the cytoplasm.

### SNHG12 upregulated the transcription of FAM83B by binding with HuR

**Fig. 7:**
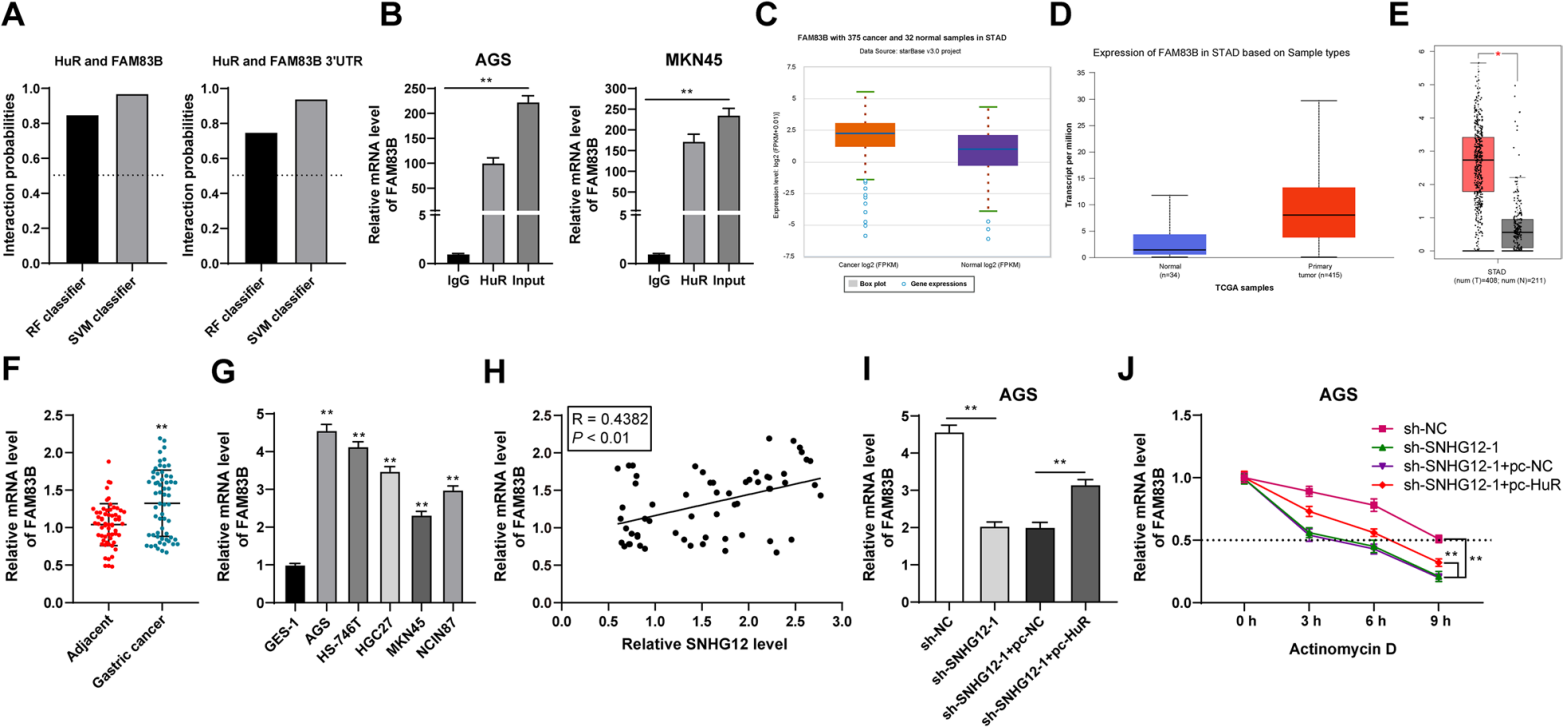
SNHG12 up-regulated the transcription of FAM83B by binding to the RNA-binding protein HuR. **A** The binding probability between HuR and FAM83B was predicted using RNA–Protein Interaction Prediction (RPISeq) database. **B** The binding between HuR and FAM83B was analyzed by RIP assay. **C**–**E** The expression of FAM83B in gastric adenocarcinoma was predicted by the Starbase, UALCAN, and GEPIA database. **F**, **G** The mRNA level of FAM83B in GC tissues and cells was detected by RT-qPCR. **H** Relevance between SNHG12 and FAM83B by Pearson. **I**, **J** mRNA level and half-life period of FAM83B in AGS cells were detected by RT-qPCR. The experiment was repeated 3 times independently. Data in panels **A**, **B**, **G**, **I** were presented as mean ± standard deviation. Comparison between data in panel F was analyzed using the t-test. Comparisons among data in panels G/I were analyzed using one-way ANOVA and comparison among data panel J was analyzed using two-way ANOVA followed by Tukey's multiple comparisons test. **p < 0.01. sh-NC: NC shRNA: sh-SNHG12: SNHG12 shRNA. pc-NC: NC pcDNA; pc-HuR: HuR pcDNA

It has been reported HuR binding with lncRNA could stabilize the expression of FAM83B [22]. It was predicted that the interacting probability of HuR with FAM83B and its 3′UTR was very high according to the prediction database (Fig. 7A). The results of the RIP assay exhibited that HuR in GC cells could bind to the FAM83B mRNA (*P* < 0.01, Fig. 7B). It was predicted that FAM83B showed high expression in gastric adenocarcinoma cells (Fig. 7C–E). The results of RT-qPCR showed that FAM83B was highly expressed in GC tissues and cells (*P* < 0.01, Fig. 7F, G) and was positively correlated with SNHG12 in GC tissues (*P* < 0.01, Fig. 7H). SNHG12 silencing or combined with HuR (PC-HuR) intervention was performed to verify that SNHG12 upregulated the transcription of FAM83B by binding to HuR. The results showed that, with the depression of SNGG12, the mRNA level of FAM83B was reduced but was increased with joint overexpression of HuR (*P* < 0.01, Fig. 7I). Then, actinomycin D was utilized to treat the intervened cells. The results showed that silencing SNHG12 could reduce the half-life period of FAM83B, while overexpression of HuR could increase the half-life period of FAM83B (*P* < 0.01, Fig. 7J). All in all, SNHG12 binding to HuR induced HuR transporting from the nucleus to the cytoplasm. HuR in the cytoplasm can bind to the mRNA of FAM83B, thereby up-regulating the transcription of FAM83B.

### Overexpression of FAM83B could reduce the inhibition of overexpression of miR-140-3p on the migration, invasion, and proliferation of GC cells

**Fig. 8:**
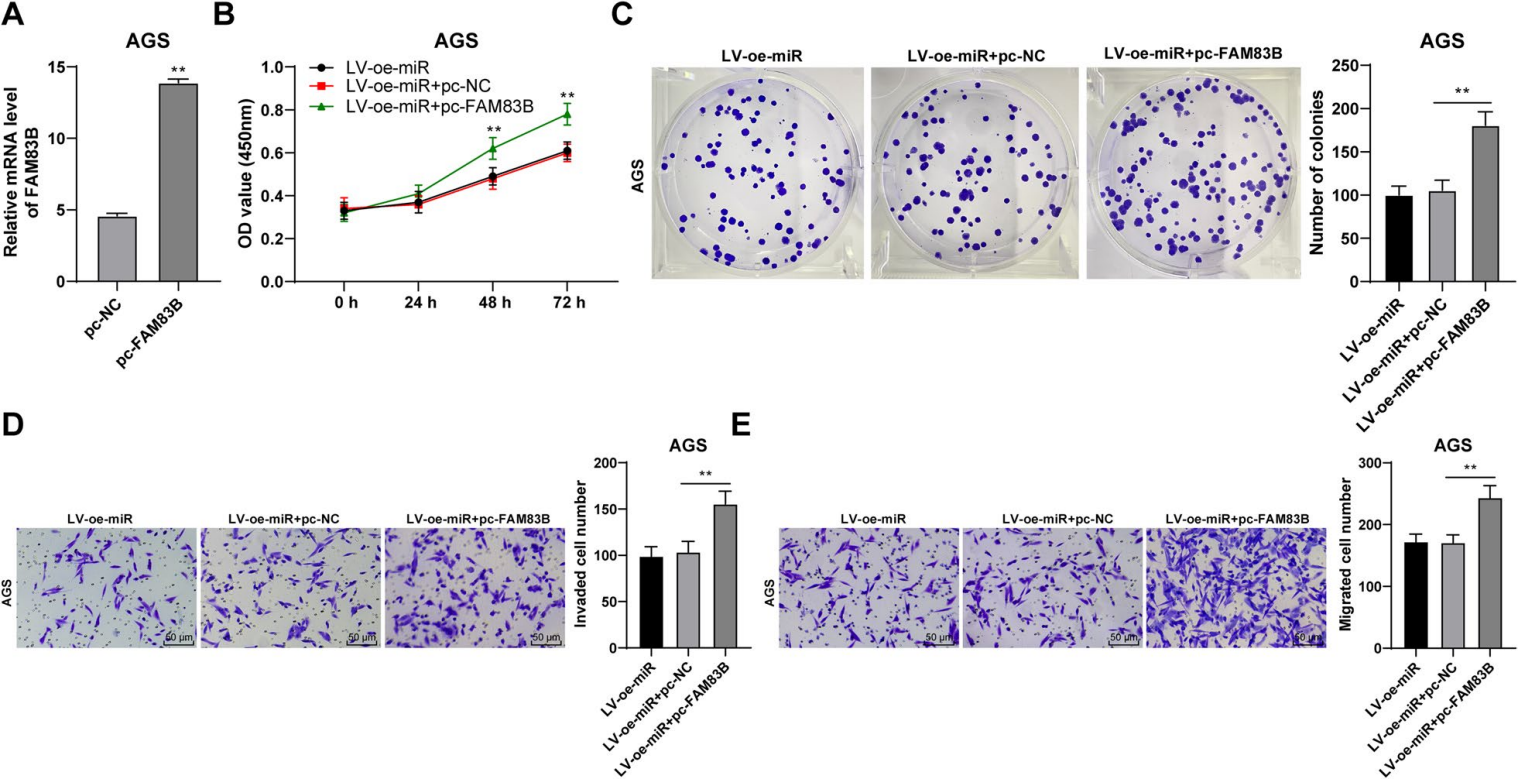
Overexpression of FAM83B reduced the inhibition of overexpression of miR-140-3p on proliferation, invasion, and migration of GC cells. FAM83B pcDNA was transfected into AGS cells. **A** mRNA level of FAM83B was in AGS cells was detected by RT-qPCR. Joint intervention with lentiviral overexpression vector of miR-140-3p was performed. The proliferation of cells was detected using CCK-8 assay (**B**) and colony formation assay (**C**). **D**, **E** The invasion and migration of cells were detected by Transwell assays. The experiment was repeated 3 times independently. Data were presented as mean ± standard deviation. Comparison between data in panel A was analyzed using the t-test. Comparisons among data in panels **C**–**E** were analyzed by using one-way ANOVA and comparison among data panel B was analyzed by using two-way ANOVA followed by Tukey's multiple comparisons test. **p < 0.01. LV-oe-miR: the lentiviral overexpression vector of miR-140-3p; pc-NC: NC pcDNA; pc-FAM83B: FAM83B pcDNA

We transfected FAM83B pcDNA into AGS cells to upregulate the intracellular post-transcriptional level of FAM83B (*P* < 0.01, Fig. 8A). Then, combined treatment with miR-140-3p lentivirus overexpression vector was performed. It was discovered that the migration, invasion, and proliferation of GC cells were remarkably increased (*P* < 0.01, Fig. 8B–E). Therefore, it was further verified that miR-140-3p regulates the migration, invasion, and proliferation of GC cells through the SNHG12/HuR/FAM83B.

### Overexpression of SNHG12 could reduce the inhibition of overexpression of miR-140-3p on growth and metastasis of gastric cancer cells in vivo

**Fig. 9:**
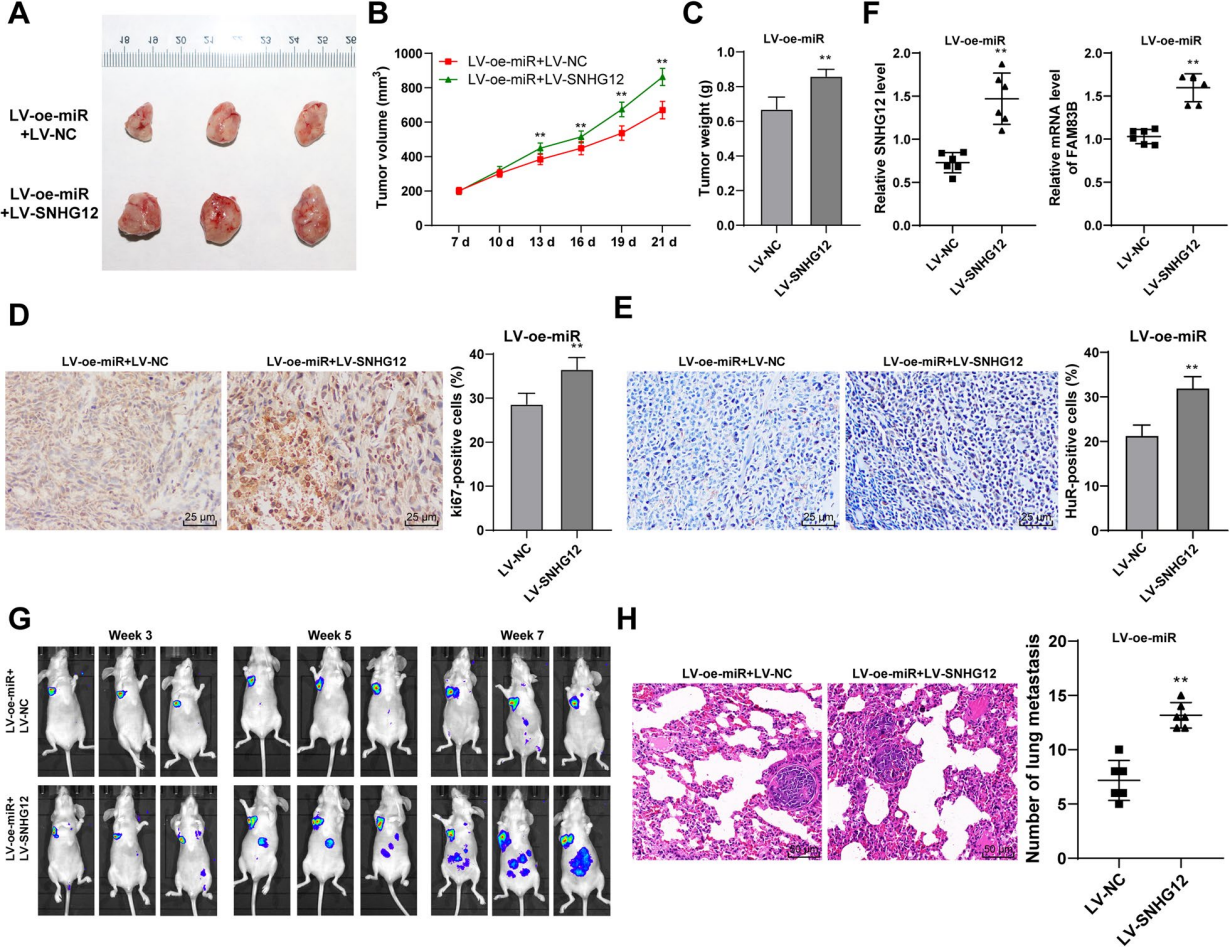
Overexpression of miR-140-3p inhibited the growth and metastasis of GC. Nude-mice transplanted tumor models were established using AGS cells with overexpression of miR-140-3p and SNHG12. **A** representative image of the transplanted tumor. **B** the volume of the tumor. **C** the weight of the tumor after euthanasia of nude mice on day 12. **D**, **E** The positive rates of ki67 and HuR in tumor tissues were detected by immunocytochemistry. **F** The level of SNHG12 and FAM83B in tumor tissues was detected by RT-qPCR. **G** The metastasis of GC was observed using vivo imaging of small animals. **H** The number of pulmonary metastasis was observed using HE staining. N = 6. Data in panels C/D/E were presented as mean ± standard deviation. Comparison between data in panels **C**–**H** was analyzed using the t-test. Comparison among data in panel **B** was analyzed by using two-way ANOVA, followed by Tukey's multiple comparisons test. **p < 0.01. LV-oe-miR: the lentiviral overexpression vector of miR-140-3p; LV-NC: The lentiviral overexpression vector of NC; LV-SNHG12: The lentiviral overexpression vector of SNHG12

The results of tumor transplantation in nude mice showed that tumor weight and volume were markedly increased after overexpression of SNHG12 (Fig. 9A–C) and the positive rate of Ki67 protein was also increased (Fig. 9D). After overexpression of SNHG12, the expression of SNHG12, the positive expression rate of HuR, and the mRNA level of FAM83B in tumor tissues were significantly increased (Fig. 9E, F). Furthermore, the results of lung metastasis exhibited that SNHG12 overexpression reduced the inhibition effect of miR-140-3p overexpression on metastasis of GC (Fig. 9G, H). Therefore, it was verified that miR-140-3p inhibits the development and metastasis of GC in vivo through the SNHG12/HuR/FAM83B.

## Discussion

GC is a highly invasive and metastatic malignancy with diagnostic difficulty and high mortality [35]. The generation and development of GC are related to the deviant expression of miRNAs [36]. miR-140-3p is commonly known for its suppression function on cells development in colorectal cancer. It also has the ability to inhibit the development of multiple solid tumors [37]. In our study, miR-140-3p directly bound to SNHG12 in GC and down-regulated the SNHG12 expression and reduced the binding of SNHG12 and HuR, thus inhibiting HuR translocating from nuclear to the cytoplasm and the binding of HuR and FAM83B, and reducing the transcription of FAM83B, and finally inhibiting the development and metastasis of GC.

Previous studies reported that miRNAs manipulate the occurrence and metastasis of GC during its progression and miR-140-3p was expressed differently in GC [30, 31]. In this study, miR-140-3p was poorly expressed in GC tissues and cells. We aimed to further explore the clinical value of miR-140-3p in GC. According to the median of miR-140-3p expression in GC tissues [20], 60 GC patients were divided into a group of high expression and a group of low expression. It was found that the miR-140-3p expression was related to the tumor size, lymph node metastasis degree, and TNM stage. The survival time of patients with low miR-140-3p expression was shorter than that of patients with high miR-140-3p expression. GC patients with low expression of miR-140-3p had shorter overall survival. It was suggested in a previous study of spinal chordoma that miR-140-3p is related to the occurrence and invasion of tumors. Furthermore, it can serve as a new predictor in the recurrence and prognosis for spinal chordoma patients [37]. Altogether, miR-140-3p is poorly expressed in GC and is related to prognosis and clinicopathologic features of GC patients.

To explore the effect of miR-140-3p on GC cells, AGS cells with relatively low miR-140-3p expression were infected with miR-140-3p overexpression vectors and miR-140-3p inhibitor was transfected to MKN45 cells with relatively high miR-140-3p expression. It was found that the migration, invasion, and proliferation of GC cells were reduced and cells with miR-140-3p inhibitor showed opposite trends after overexpression of miR-140-3p. A study indicated that overexpression of miR-140-3p remarkably inhibited the migration, invasion, and proliferation of cutaneous melanoma cells [38]. All in all, overexpression of miR-140-3p inhibited the proliferation, invasion, and migration of GC cells. Furthermore, we applied AGS cells with stable overexpression of miR-140-3p to establish the xenograft tumor model and lung metastatic model in nude mice. We found that tumor growth was inhibited and tumor weight was significantly reduced, and the number of lung metastasis markedly reduced after overexpression of miR-140-3p. It has been identified that miR-140-3p can serve as a suppressor in several malignancies. Binding to PD-L1, miR-140-3p can serve as a suppressor in tumor development in *vivo* via inhibition of the PIAK/AKT pathway [39]. Hence, overexpression of miR-140-3p may inhibit the development and metastasis of GC.

Then, we continued to explore the downstream mechanism of miR-140-3p. It has been identified that miRNA can directly bind to lncRNA SNHG12 to regulate the stability of SNHG12, thereby regulating the expression of SNHG12 [10, 11]. The binding between miR-140-3p and SNHG12 was confirmed using dual-luciferase assay and RIP assay. It has been proved in a previous study that SNHG12 shows high expression in gastric adenocarcinoma, and the survival time of GC patients with high SNHG12 expression was markedly shorter than that of patients with low SNHG12 expression. A previous finding demonstrated that SNHG12 serves as a potential therapeutic target and prognostic marker for GC [13]. It was found that after directly binding with miR-140-3p in GC, SNHG12 can be depressed. To verify the role of SNHG12 in miR-140-3p regulating GC cells, AGS cells were infected with SNHG12 overexpressing vector and then treated with miR-140-3p lentivirus overexpression vector. the migration, invasion, and proliferation of GC cells were remarkably increased. We concluded that SNHG12 overexpression could reduce the inhibition of overexpression of miR-140-3p on the migration, invasion, and proliferation of GC cells and miR-140-3p inhibited the expression of SNHG12 to regulate the migration and proliferation of GC cells. The in vivo experiments further validated the in vitro results. A previous study indicated that inhibition of SNHG12 suppresses GC cells proliferation and migration, and thus suggests that SNHG12 interaction may be used as a promising target for GC treatment [40]. In conclusion, overexpression of SNHG12 can reduce the inhibition of overexpression of miR-140-3p the migration, invasion and proliferation, and the development and metastasis of GC cells.

Next, the downstream mechanism of SNHG12 was further explored. It was exhibited in the result of a subcellular fractionation assay and RNA FISH assay that SNHG12 was located chiefly in the cytoplasm of GC cells. RIP assay verified that SNHG12 in GC cells was able to bind to HuR. SNHG12 can bind to HuR [18]. HuR shows high expression in GC tissues and cells [41]. Next, we transfected shRNA of SNHG12 (sh-SNHG12) into AGS cells. After the SNHG12 knockdown, HuR expression in the cells was markedly decreased, which in the nucleus was increased. Overall, SNHG12 bound to the RNA-binding protein HuR and then induced HuR translocating from the nuclear to the cytoplasm.

It has been discovered that FAM83B can be upregulated in different kinds of cancer samples and has the potential to be new targets [42]. In a previous study, it has been confirmed that HuR binding to SNHG12 can stabilize the expression of FAM83B [22]. This study elicited that the HuR in GC cells could bind to the FAM83B mRNA and FAM83B showed high expression in GC cells and tissues. It was also positively correlated with SNHG12 in GC tissues. SNHG12 silencing or combined with HuR overexpression was performed to verify that SNHG12 upregulated the transcription of FAM83B by binding to HuR. The results showed that, with the depression of SNGG12, the mRNA level of FAM83B was reduced but was increased with joint overexpression of HuR. Moreover, FAM83B pcDNA was transfected into AGS cells combined with the treatment of miR-140-3p lentivirus overexpression vector. It was found that the migration, invasion, and proliferation of GC cells were significantly increased. A previous study found that overexpression of FAM83B can promote the proliferation of lung cancer cells [43]. There is little study on the mechanism of FAM83B on the cellular function of GC cells. Our results initially demonstrated that FAM83B overexpression can reduce the inhibition of miR-140-3p overexpression on proliferation, invasion, and migration of GC cells.

## Conclusions

**Fig. 10:**
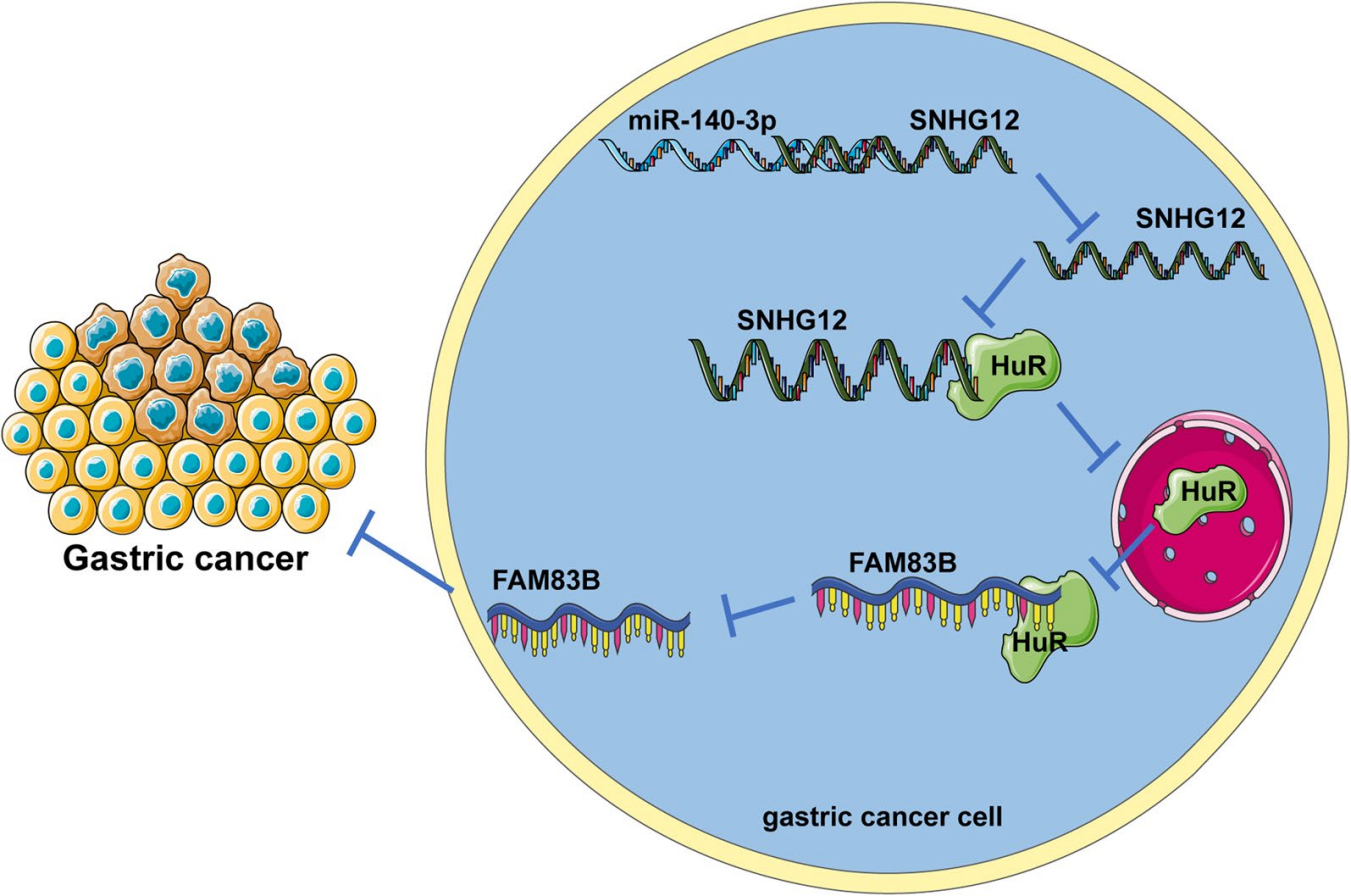
Effects and mechanisms of miR-140-3p on the growth and metastasis of GC. First, miR-140-3p directly bound to SNHG12 in GC and thus down-regulated the expression of SNHG12and reduced the binding of SNHG12 and HuR, inhibiting the transportation of HuR from the nuclei to the cytoplasm. Therefore, the binding of HuR and FAM83B mRNA was inhibited and FAM83B mRNA’s stability was reduced, and the transcription of FAM83B was down-regulated. Furthermore, the proliferation, invasion, and migration of GC cells were inhibited and the growth and metastasis of GC were inhibited

In conclusion, miR-140-3p directly bound to SNHG12 in GC and down-regulated the expression of SNHG12, reduced the binding of SNHG12 and HuR, inhibited the nuclear transportation and the binding between HuR and mRNA of FAM83B, thereby downregulating the transcription of FAM83B, and eventually, the growth and metastasis of GC were inhibited (Fig. 10). In general, the downstream mechanism of miRNAs is usually to explore the target genes downstream of miRNAs, and our research mechanism is that miRNA affects the expression of lncRNA by affecting the stability of lncRNA. Furthermore, to study the relationship between lncRNA, miRNA, and mRNA, ceRNA mechanism is generally used, that is, miRNA and mRNA competitively bind to lncRNA to affect mRNA expression, but our research mechanism is that miRNA and RNA-binding protein competitively bind to lncRNA to affect mRNA expression. These are the novelties of our study. However, there are limitations in this study. This study failed to explore more relations between miR-140-3p and SNHG12. Whether there is a ceRNA mechanism between miR-140-3p and SNHG12 remains to be explored. In addition, whether SNHG12 could bind to other RNA-binding proteins needs to be further investigated. Furthermore, the downstream mechanism of SNHG12 binding to HUR still needs to be improved. In the future, the ceRNA mechanism between miR-140-3p and SNHG12 shall be studied and the mechanism of SNHG12 binding to other RNA-binding proteins shall be further explored to provide new theoretical knowledge for the treatment of GC.

## Data Availability

The data that support this study are available from the corresponding author upon reasonable request.

## Abbreviations

GC: Gastric cancer
RIP: RNA immunoprecipitation
miRNAs: MicroRNAs
SNHG12: Small nucleolar RNA host gene 12
HuR: Human antigen R
RNA FISH: RNA Fluorescence in situ hybridization
FAM83B: Family with sequence similarity 83 member B
HE: Hematoxylin and eosin
CCK-8: Cell counting kit-8
PBS: Phosphate buffered saline
qRT-PCR: Quantitative real-time polymerase chain reaction
DAB: Diaminobenzidine
RIPA: Radioimmunoprecipitation
TNM: Tumor lymph node metastasis
GAPDH: Reduced glyceraldehyde-phosphate dehydrogenase
ANOVA: Analysis of variance
